# Hydrothermal Synthesis of Heterostructured g-C_3_N_4_/Ag–TiO_2_ Nanocomposites for Enhanced Photocatalytic Degradation of Organic Pollutants

**DOI:** 10.3390/ma16155497

**Published:** 2023-08-07

**Authors:** Agidew Sewnet, Esayas Alemayehu, Mulualem Abebe, Dhakshnamoorthy Mani, Sabu Thomas, Bernd Lennartz

**Affiliations:** 1 Faculty of Materials Science and Engineering, Jimma Institute of Technology, Jimma University, Jimma P.O. Box 378, Ethiopia; agidewsewnet@gmail.com (A.S.); muabeme@gmail.com (M.A.); m.dhakshnamoorthy@gmail.com (D.M.); 2 Department of Physics, College of Natural and Computational Science, Bonga University, Bonga P.O. Box 334, Ethiopia; 3 Faculty of Civil and Environmental Engineering, Jimma University, Jimma P.O. Box 378, Ethiopia; 4 Africa Center of Excellence for Water Management, Addis Ababa University, Addis Ababa P.O. Box 1176, Ethiopia; 5 International and Inter University Centre for Nanoscience and Nanotechnology, Mahatma Gandhi University, Kottayam 686560, India; sabuthomas@mgu.ac.in; 6 Faculty of Agricultural and Environmental Sciences, University of Rostock, Justus-Von-Liebig-Weg 6, 18059 Rostock, Germany

**Keywords:** g-C_3_N_4_/Ag–TiO_2_, hydrothermal synthesis, heterogeneous photocatalysis, rhodamine B, visible LED light irradiation

## Abstract

In this study, heterostructured g-C_3_N_4_/Ag–TiO_2_ nanocomposites were successfully fabricated using an easily accessible hydrothermal route. Various analytical tools were employed to investigate the surface morphology, crystal structure, specific surface area, and optical properties of as-synthesized samples. XRD and TEM characterization results provided evidence of the successful fabrication of the ternary g-C_3_N_4_/Ag–TiO_2_ heterostructured nanocomposite. The heterostructured g-C_3_N_4_/Ag–TiO_2_ nanocomposite exhibited the best degradation efficiency of 98.04% against rhodamine B (RhB) within 180 min under visible LED light irradiation. The g-C_3_N_4_/Ag–TiO_2_ nanocomposite exhibited an apparent reaction rate constant 13.16, 4.7, and 1.33 times higher than that of TiO_2_, Ag–TiO_2_, and g-C_3_N_4_, respectively. The g-C_3_N_4_/Ag–TiO_2_ ternary composite demonstrated higher photocatalytic activity than pristine TiO_2_ and binary Ag–TiO_2_ photocatalysts for the degradation of RhB under visible LED light irradiation. The improved photocatalytic performance of the g-C_3_N_4_/Ag–TiO_2_ nanocomposite can be attributed to the formation of an excellent heterostructure between TiO_2_ and g-C_3_N_4_ as well as the incorporation of Ag nanoparticles, which promoted efficient charge carrier separation and transfer and suppressed the rate of recombination. Therefore, this study presents the development of heterostructured g-C_3_N_4_/Ag–TiO_2_ nanocomposites that exhibit excellent photocatalytic performance for the efficient degradation of harmful organic pollutants in wastewater, making them promising candidates for environmental remediation.

## 1. Introduction

Nowadays, organic contaminants have become major sources of water pollution worldwide due to population growth and rapid agricultural and industrial development [1]. Modern commercial dyes are a major category of organic pollutants known for their robust structural and color stability owing to their high degree of aromaticity and extensively conjugated chromophores. With widespread use in diverse industries, such as food, cosmetics, leather, plastics, printing, and textiles, these dyes pose a health risk to humans and ecological systems if unintentionally released into the environment [2,3]. Furthermore, the discharge of these toxic and carcinogenic synthetic colors into water bodies could obstruct sunlight penetration, which is harmful to natural aquatic activities that involve photosynthesis and other biodegradation processes. These hazardous pollutants seriously harm human health and the environment if they are not effectively removed [1,4]. Therefore, various conventional methods have been used for wastewater treatment, such as filtration, adsorption, advanced oxidation, coagulation, sedimentation, disinfection, reverse osmosis, and biological processes [1,5]. However, these conventional methods have several limitations, including low efficiency, high chemical costs, difficult preparation, time consumption, a limited visible light absorption range, and the generation of secondary waste [1,6]. Therefore, it is imperative to develop efficient, cost-effective, and sustainable wastewater treatment technologies to eliminate hazardous organic pollutants [5]. Since the pioneering work of Fujishima and Honda in 1972, heterogeneous photocatalysis has attracted tremendous attention as an advanced oxidation process for the elimination of pollutants in wastewater [7]. Therefore, photocatalysis is a sustainable, cost-effective, green, and clean technology for the decomposition of organic pollutants in wastewater into H_2_O, CO_2_, and inorganic minerals [8]. Photocatalysts use solar energy to promote electrons from the valence band to the conduction band, leading to the generation of electron–hole pairs, which then initiate reactions with water and oxygen molecules or hydroxyl groups. Therefore, superoxide anions (^•^O_2_^−^) and hydroxyl radicals (^•^OH) are produced, and these reactive oxygen species (ROS) actively participate in photochemical redox reactions throughout the photocatalytic process [9]. Moreover, holes may react with hydroxyl ions (OH^−^) or water molecules to produce hydroxyl radicals (^•^OH), and they may directly participate in the oxidative decomposition of pollutants [8].

TiO_2_ is the most widely used oxide-based semiconductor photocatalyst for the elimination of harmful organic pollutants because of its intriguing properties such as low cost, high stability, and excellent optical properties [10,11]. However, owing to its high energy bandgap (3.2 eV), TiO_2_ can only be active in the UV light region, which accounts for less than 5% of the total solar energy, leading to limited utilization of solar energy. Additionally, the poor photocatalytic efficacy of TiO_2_ can be attributed to the fast recombination of photoinduced charge carriers [12]. Many researchers have explored various strategies to overcome the limitations of TiO_2_. Among them, many studies have focused on noble metal deposition and semiconductor coupling for the formation of heterojunctions [9]. Recently, noble metal deposition on TiO_2_ photocatalysts has attracted extensive research attention [10,12,13]. The surface plasmon resonance effect of noble metals, such as Ag, Au, Pt, and Pd, can improve charge carrier separation and transfer and visible light absorption, leading to enhanced photocatalytic efficiency [5,14]. Moreover, the migration of electrons from the conduction band of TiO_2_ to noble metals is facilitated by the creation of a Schottky barrier at the interface, promoting the separation of photoinduced charge carriers and retarding the recombination of electron–hole pairs, all of which contribute to the overall enhancement of the photocatalytic performance [11,15]. Among the different noble metals, silver (Ag) remains the primary choice because of its high stability, low cost, and facile preparation [16]. In addition, Ag possesses vacant orbitals that can serve as active sites and electron acceptors, facilitating the movement of electrons in composite materials, and its surface plasmon resonance effect can significantly broaden the visible light absorption range [15,17]. Therefore, researchers have shown significant interest in Ag deposition on TiO_2_ because of its potential to enhance the photoactivity of TiO_2_ [13,18,19,20]. The key role of Ag in Ag-doped TiO_2_ nanoparticles is to enhance their photocatalytic activity through mechanisms such as electron trapping, improving the visible light absorption range, and modifying the surface properties, leading to improved electron–hole separation and greater surface electron excitation [13]. Although the deposition of Ag can decrease the rate of photoinduced charge carrier recombination, it is not sufficient to improve TiO_2_ photocatalytic performance at a broad range of visible-light absorption. Therefore, the deposition of plasmonic Ag metal, in tandem with coupling with small-bandgap semiconductors having suitable energy bandgap positions, can create a heterojunction that further boosts the photocatalytic efficiency of TiO_2_ [5]. In recent years, the coupling of TiO_2_ and g-C_3_N_4_ has gained considerable attention owing to their highly compatible band positions [21,22,23,24,25,26]. A heterojunction formed between TiO_2_ and g-C_3_N_4_ reduces the recombination rate of charge carriers and improves photocatalytic activity [1]. Therefore, g-C_3_N_4_ is considered to be the best candidate for coupling with TiO_2_ to construct heterojunctions because of its suitable band position, chemical stability, and low cost [27]. Graphitic carbon nitride (g-C_3_N_4_) is an n-type polymeric semiconductor that has been extensively studied owing to its potential use in the elimination of organic pollutants. It is cost-effective, non-toxic, eco-friendly, easy to synthesize, and has a mid-energy bandgap (2.7 eV) as well as strong physicochemical stability [6]. Despite its many advantages, bulk g-C_3_N_4_ exhibits weak photocatalytic performance owing to factors such as fast recombination of photoexcited charge carriers, limited visible light absorption range, and low specific surface area [28]. Researchers have found that modifying the photocatalytic properties of g-C_3_N_4_ is as important as modifying TiO_2_, and the synergistic effect of Ag deposition on TiO_2_ and coupling with g-C_3_N_4_ can lead to enhanced photocatalytic activity of the ternary composites [29,30,31,32,33,34,35,36]. In particular, heterostructured g-C_3_N_4_/Ag–TiO_2_ nanocomposites have emerged as a promising area of research in the field of photocatalytic degradation of organic pollutants and have been extensively studied due to their remarkable visible-light-driven photocatalytic performance. Therefore, many strategies have been used to fabricate heterostructured g-C_3_N_4_/Ag–TiO_2_ nanocomposites, including the microwave-assisted approach [37], freeze-drying route [9], hydrothermal method [36,38], calcination [39,40], physical mixing-calcination method [41], physical mixing [42,43], chemical reduction methods, and so on [11].

Most recent research findings on g-C_3_N_4_/Ag–TiO_2_ nanocomposites have used xenon lamps with cutoff filters [11,14,15,17,42,43,44] as visible light sources. However, they have various drawbacks, such as being hazardous, having a short lifespan, being overheated quickly, being expensive, and posing difficulties in handling [6]. However, LED lamps are non-toxic and energy-efficient alternatives to conventional lamps because they convert more energy into light and do not generate excessive heat. Furthermore, LED lamps are cost-effective, durable, and emit only the wavelengths required to save energy [45]. As part of green and sustainable chemistry, this study used an energy-saving 50 W LED lamp to measure the photocatalytic degradation efficiency of the as-synthesized samples.

In this study, a heterostructured g-C_3_N_4_/Ag–TiO_2_ nanocomposite was successfully fabricated using an easily accessible hydrothermal technique, followed by calcination treatment. First, a simple sol-gel synthesis route was used to prepare pristine TiO_2_ and 3 mol% Ag-doped TiO_2_ nanoparticles. Second, g-C_3_N_4_ nanosheets were synthesized using the single-step calcination of a mixture of urea and thiourea at 600 °C in an air medium. Finally, a facile one-step hydrothermal technique and calcination were used to prepare a g-C_3_N_4_/Ag–TiO_2_ nanocomposite using a desired weight ratio of 3:1 from the fabricated g-C_3_N_4_ nanosheet to 3 mol% Ag–TiO_2_ nanoparticles. The structural, morphological, and optical properties and specific surface area of the prepared materials were characterized by field-emission scanning electron microscopy (FE-SEM), high-resolution transmission electron microscopy (HR-TEM), X-ray diffraction (XRD), Fourier transform infrared spectroscopy (FTIR), Brunauer–Emmett–Teller (BET) nitrogen adsorption/desorption technique (BET), photoluminescence (PL), and UV/vis diffuse reflectance spectroscopy (DRS). In addition, the photocatalytic performance of the prepared pristine TiO_2_, binary Ag–TiO_2_ nanoparticles, g-C_3_N_4_ nanosheets_,_ and g-C_3_N_4_/Ag–TiO_2_ nanocomposites was examined by assessing their ability to degrade rhodamine B (RhB) upon illumination by energy-saving visible LED light.

## 2. Materials and Methods

### 2.1. Materials

In this study, Ag-doped TiO_2_ nanoparticles were prepared using several reagents, including titanium tetra isopropoxide (C_12_H_28_O_4_Ti, 97%, Sigma-Aldrich, St. Louis, MO, USA), silver nitrate (AgNO_3_, 99%, Merck, Boston, MA, USA), glacial acetic acid (CH_3_COOH, 99%, Merck), nitric acid (HNO_3_, 69%, Merck), and absolute ethanol (C_2_H_5_OH, Sigma-Aldrich). Moreover, analytical-grade urea (CH_4_N_2_O, 99.5%, Merck) and thiourea (CH_4_N_2_S, 99%, Merck) were used to synthesize g-C_3_N_4_ nanosheets. Fabricated Ag–TiO_2_ nanoparticles and g-C_3_N_4_ nanosheets were used to synthesize heterostructured g-C_3_N_4_/Ag–TiO_2_ nanocomposites. All the chemicals and reagents were used as received without further purification. Distilled water was used in all experiments.

### 2.2. Synthesis

#### 2.2.1. Preparation of g-C_3_N_4_ Samples

g-C_3_N_4_ nanosheets were prepared by one-step calcination of a mixture of urea and thiourea in a muffle furnace under an air atmosphere. Typically, specific amounts of urea and thiourea were carefully weighed and placed into a 50 mL ceramic crucible with a lid, which was then wrapped with aluminum foil paper and subjected to calcination at 600 °C for 3 h. The resulting product was cooled to room temperature and carefully ground into a fine powder.

#### 2.2.2. Preparation of Ag–TiO_2_ Nanoparticles

Ag-doped TiO_2_ (Ag–TiO_2_) nanoparticles were prepared using a sol-gel method. First, solution A was obtained by dissolving 22 mL of titanium isopropoxide (TTIP) in 150 mL of ethanol, followed by constant stirring for 45 min. In solution B, 3 mol% of silver nitrate was dissolved in 40 mL of distilled water with the addition of 5 mL of glacial acetic acid. Solution B was then slowly dripped into solution A and vigorously stirred. The resulting milky sol was constantly stirred for 2 h while carefully adjusting the pH value to 2–3 by adding 2 mL of nitric acid. Subsequently, the white precipitate obtained from the sol was allowed to settle for 4 days at room temperature, after which the solid gel was collected and washed several times with distilled water by centrifugation at 4000 rpm for 10 min. The washed samples were dried at 80 °C for 16 h in a vacuum oven. The resulting products were ground and calcined at 400 °C for 4 h. Finally, the samples were ground again and used for characterization. Similarly, pristine TiO_2_ nanoparticles were synthesized under the same experimental conditions without the addition of AgNO_3_ as a precursor of Ag.

#### 2.2.3. Fabrication of g-C_3_N_4_/Ag–TiO_2_ Nanocomposite

A facile hydrothermal method followed by calcination was used to fabricate a heterostructured g-C_3_N_4_/Ag–TiO_2_ nanocomposite with a weight ratio of 3:1 of fabricated g-C_3_N_4_ nanosheets and 3% Ag–TiO_2_ nanoparticles, respectively. The process involves dissolving 0.3 g of g-C_3_N_4_ nanosheets in 100 mL of distilled water by ultrasonication for 1 h and then adding 0.1 g of 3% Ag–TiO_2_ nanoparticles to the solution, followed by vigorous stirring for 2 h. In addition, a small amount of hydrofluoric acid was added continuously during stirring to control nanocrystal formation. The resulting solution was transferred to a 100 mL stainless-steel autoclave and maintained at 180 °C for 6 h. Following hydrothermal treatment, the mixture was cooled to room temperature and subsequently washed several times with distilled water. Finally, the resulting sample was dried at 80 °C for 15 h and then ground into an ultrafine powder.

#### 2.2.4. Characterization

The crystal structures of the as-synthesized samples were analyzed using X-ray powder diffraction (PanAlyticals, Almelo, The Netherlands) with Cu-Kα radiation (λ = 1.5406 Å) operating at 40 kV and 15 mA. The diffraction patterns were measured over a range of 10–70°, with a step width of 0.01° and a scanning rate of 10°/min. The functional groups and chemical structures of the samples were analyzed using FTIR spectroscopy (IR Tracer-100, Shimadzu, Kyoto, Japan) within the wavenumber range of 600–3600 cm^−1^. The bandgap energies of the samples were analyzed using UV/vis diffuse reflectance spectroscopy (UV-2600, Shimadzu, Kyoto, Japan) with barium sulfate as the background substance within the wavelength range of 300–700 nm. The separation of photoinduced electron–hole pairs in the as-prepared samples was examined using PL spectrophotometer (RF-6000, Shimadzu, Kyoto, Japan) with an excitation wavelength of 360 nm. Field-emission scanning electron microscopy (FE-SEM, MAIA3 XMH, TESCAN BRONO s.r.o, Brono, Czech) and high-resolution transmission electron microscopy (HR-TEM, JEM-2100F, JEOL, Tokyo, Japan) were used to investigate the morphology of the samples at an accelerating voltage of 200 kV. The N_2_ adsorption–desorption isotherms and Brunauer–Emmett–Teller specific surface area (S_BET_) analyses were performed using a Micromeritics ASAP 3000 (Micromeritics, Norcross, GA, USA) equipped with a nitrogen adsorption device. Additionally, the Barrett–Joyner–Halenda (BJH) method was used to determine the pore size distribution and volume curves of the as-synthesized samples.

#### 2.2.5. Photocatalytic Activity Testing

The photocatalytic activities of as-prepared photocatalysts were determined by assessing their ability to degrade rhodamine B (RhB) using a 50 W crystal white LED lamp (5000 lm, 6500 k, 220–240 V) as a visible light source (Phillips, Kolkata, India). A photolysis experiment was conducted on an aqueous RhB solution under visible LED light illumination for 180 min. Adsorption and photocatalytic experiments were performed by dispersing 50 mg of each photocatalyst in 100 mL of a 10 mg/L RhB aqueous solution. A dark adsorption experiment was conducted for 60 min to establish the adsorption–desorption equilibrium between RhB and the photocatalysts. Throughout the experiments, vigorous magnetic stirring was applied. At specific time intervals during each experiment, 3 mL aliquots were withdrawn from the solution and subjected to centrifugation to separate the photocatalyst. The absorbance of RhB was measured at its maximum absorption wavelength (λ = 554 nm) using a UV/vis spectrophotometer (2450, Shimadzu, Kyoto, Japan). The rate of RhB dye degradation (η) in aqueous solution was calculated using the following equation [46,47]:

(1) $$\eta =\frac{A_{0}-A}{A_{0}}\times 100\%$$

where A_0_ and A refer to the initial absorbance and absorbance at a specific irradiation time t, respectively.

A pseudo-first-order kinetic model was utilized to determine the apparent degradation rate constant (k_app_) of RhB dye over g-C_3_N_4_/Ag–TiO_2_ composites [11,47]:

(2) $$\;ln\frac{A_{0}}{A}=k_{app}t$$

where k_app_ (min^−1^) is the rate constant and t (min) is the specific time interval of irradiation.

## 3. Results and Discussion

### 3.1. XRD Analysis

**Figure 1:**
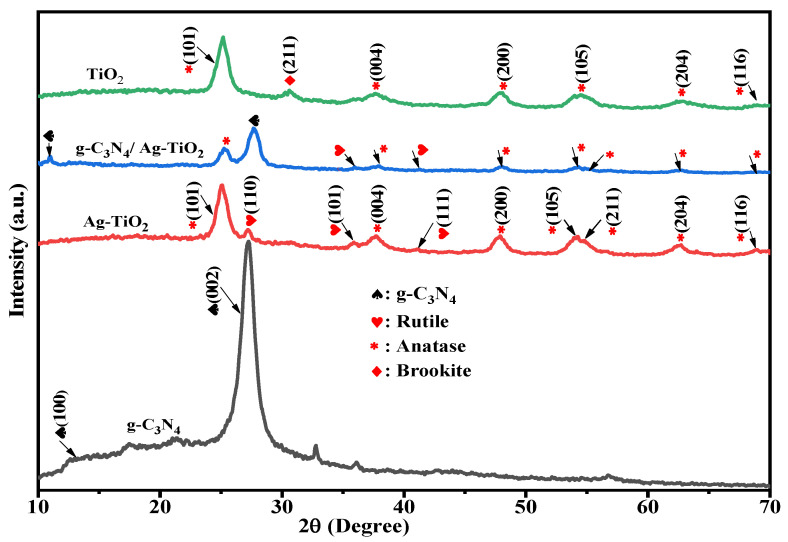
XRD patterns of as-prepared samples.

The crystal structures of the as-prepared samples, including pristine TiO_2_, Ag–TiO_2_, g-C_3_N_4_, and g-C_3_N_4_/Ag–TiO_2_, were investigated using X-ray powder diffraction (XRD), and the results are shown in Figure 1. The XRD patterns of g-C_3_N_4_ revealed the presence of two characteristic peaks at around 13.1° (100) and 27.2° (002), which correspond to in-plane repeating tri-s-triazine units and interplanar stacking of conjugated aromatic units, respectively [6,48]. The absence of a characteristic Ag peak in the composites, as confirmed by the XRD spectra, can be attributed to the low amount of Ag or its high dispersion [38,41,42]. The diffraction peaks of pristine TiO_2_ and Ag–TiO_2_ nanoparticles are predominantly in the anatase phase (ICSD File No. 076173) and located at 2θ positions of around 25.356° (101), 37.847° (004), 48.145° (200), 53.974° (105), 55.186° (211), 62.812° (204), and 68.879° (116). In addition to the characteristic anatase phase peaks, the Ag–TiO_2_ nanoparticles also exhibited a rutile phase, with diffraction peaks located at 2θ of 27.4392° (110), 306.0847° (101), and 3541.2744° (111) (ICSD File No. 088627). The pristine TiO_2_ sample was primarily composed of the anatase phase, with a minor brookite phase at 2θ of 30.68°, corresponding to the (121) crystal plane, which indicated consistency with previous findings [49]. The XRD patterns of the g-C_3_N_4_/Ag–TiO_2_ nanocomposite contained diffraction peaks arising from both Ag–TiO_2_ and g-C_3_N_4_, thereby confirming the successful formation of the composite. Moreover, a higher g-C_3_N_4_ content was found to be capable of intensifying the recognizable peak at approximately 27.6°, resulting in a minor peak position shift in the composite [11]. Both anatase and rutile TiO_2_ diffraction peaks were observed for the g-C_3_N_4_/Ag–TiO_2_ composite [42,50].

The average crystallite size (D) of the as-synthesized photocatalysts was estimated using the Scherrer equation [21]:

(3) $$D=\frac{K\lambda }{\beta cos\theta }$$

where K is a constant (0.94), λ is the X-ray wavelength (0.15406 nm), β is the full width at half maximum (FWHM), and θ is the diffraction angle. As calculated from the Scherrer equation, the average crystallite sizes of the as-synthesized photocatalysts, including pure TiO_2_, Ag–TiO_2_, g-C_3_N_4_, and g-C_3_N_4_/Ag–TiO_2_, were approximately 5.2, 9.1, 4.3, and 7.6 nm, respectively.

### 3.2. Optical Properties Analysis

#### 3.2.1. UV/vis DRS Analysis

**Figure 2:**
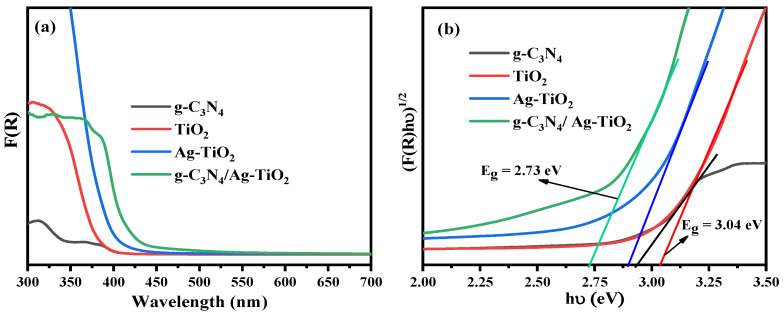
UV/vis diffuse reflectance spectra of the as-synthesized samples (**a**,**b**) the corresponding modified Tauc plot using the Kubelka–Munk function.

The light absorption properties and bandgap energies of all synthesized samples, including TiO_2_, g-C_3_N_4_, Ag–TiO_2_, and g-C_3_N_4_/Ag–TiO_2_, were analyzed using UV/vis diffuse reflectance spectroscopy (DRS), as shown in Figure 2.

As shown in Figure 2b, the bandgap energies of the Ag–TiO_2_ and g-C_3_N_4_/Ag–TiO_2_ composites are narrower than those of the pristine TiO_2_ and g-C_3_N_4_ samples. The absorption edges of the g-C_3_N_4_/Ag–TiO_2_ nanocomposite exhibited a considerable redshift toward higher wavelengths, indicating a significant improvement in the visible light absorption of the composites, as shown in Figure 2a [11,50]. This may be due to the surface plasmon resonance effect of Ag, which is deposited on the surface of the TiO_2_ nanoparticles, and the formation of heterostructures in the composites [17]. The bandgap energy (E_g_) of the as-synthesized samples was calculated using a modified Tauc equation with the Kubelka–Munk function:

(4) $$\left(F(R){h\upsilon )}^{\frac{1}{2}}=A(h\upsilon -E_{g}\right)$$

where F(R) = (1 − R)^2^/2R, F(R) is the Kubelka–Munk function, R is the reflectance, hυ is the photon energy, and A is a constant [11]. The bandgap energy can be estimated by determining the point at which the linear trendline on the plot of (F(R)hυ)^1/2^ versus (hυ) intersects the hυ-axis [11,42]. The bandgap energies of TiO_2_, Ag–TiO_2_, g-C_3_N_4_, and g-C_3_N_4/_Ag–TiO_2_ photocatalysts were calculated to be 3.04, 2.89, 2.94, and 2.73 eV, respectively. Compared to pristine TiO_2_ and g-C_3_N_4_, Ag–TiO_2_ shows a slight red shift in the absorption edge [9]. Furthermore, coupling g-C_3_N_4_ with Ag–TiO_2_ shows a significant red shift compared to TiO_2_, Ag–TiO_2_, and g-C_3_N_4_, which allows for enhanced visible-light absorption [48]. Therefore, the synergetic effect of coupling g-C_3_N_4_ and loading Ag on TiO_2_ ameliorated the separation and transportation of photogenerated charge carriers, leading to an overall increase in photocatalytic performance [9,51].

#### 3.2.2. PL Analysis 

**Figure 3:**
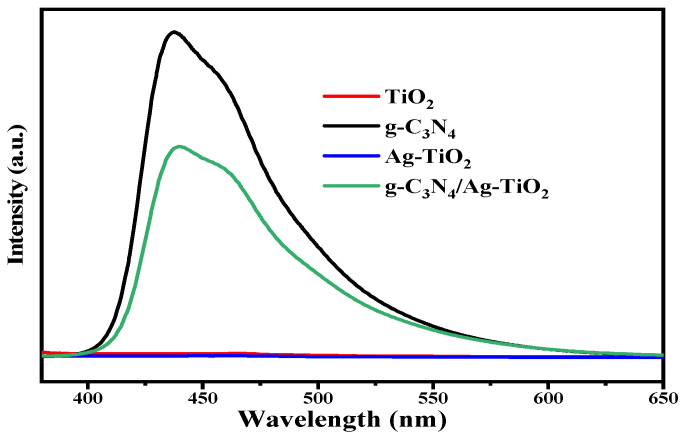
PL spectra of TiO_2_, Ag–TiO_2_, g-C_3_N_4_, and g-C_3_N_4_/Ag–TiO_2_ samples.

Photoluminescence (PL) analysis was used to study the separation of photoinduced electron–hole pairs in the as-synthesized samples [43]. Figure 3 shows the PL spectra of pristine TiO_2_, Ag–TiO_2_, g-C_3_N_4_, and g-C_3_N_4_/Ag–TiO_2_ samples obtained at an excitation wavelength of 360 nm. Both g-C_3_N_4_ and g-C_3_N_4_/Ag–TiO_2_ displayed main emission peaks at approximately 440 nm, as shown in Figure 3. A higher PL intensity was observed for g-C_3_N_4_ compared to g-C_3_N_4_/Ag–TiO_2_, indicating faster recombination of photoinduced electron–hole pairs in g-C_3_N_4_ [36]. The PL spectra of pristine TiO_2_ and Ag–TiO_2_ nanoparticles did not exhibit any significant emission peaks, indicating that they had a lower rate of recombination of photogenerated electron–hole pairs [50]. The reduced PL emission peak of the g-C_3_N_4_/Ag–TiO_2_ composite compared to g-C_3_N_4_ can be attributed to a lower rate of recombination of photogenerated electron–hole pairs, which may be due to the formation of a heterostructure between g-C_3_N_4_ and Ag–TiO_2_ [42,43].

#### 3.2.3. FTIR Analysis

**Figure 4:**
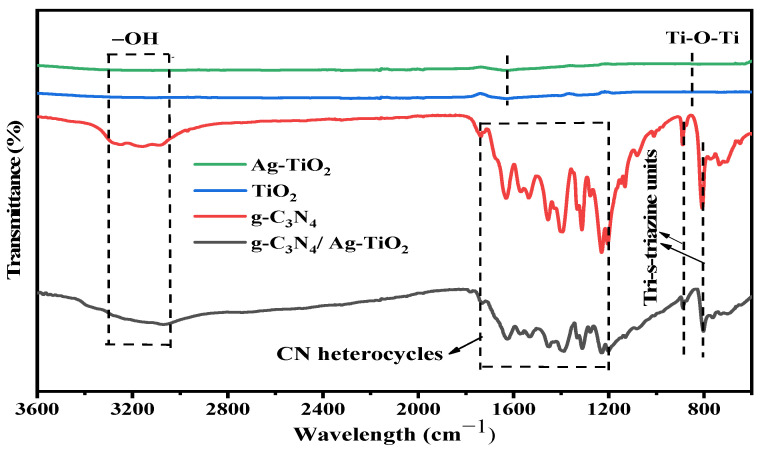
FTIR spectra of as-synthesized samples.

Figure 4 shows the results of the FTIR analysis conducted to identify the functional groups and chemical structures of all-prepared photocatalysts.

The stretching modes of C–N and C=N heterocycles were observed at 1200–1740 cm^−1^ in the g-C_3_N_4_ samples [6,52]. In the g-C_3_N_4_ samples, peaks observed at around 1458, 1560, and 1630 cm^−1^ can be attributed to C=N heterocycles, and the stretching mode of C=O was detected at 1740 cm^−1^. Additionally, the FTIR peaks at 1230, 1319, and 1400 cm^−1^ represent the aromatic C–N stretching mode, whereas the peaks at 805 and 885 cm^−1^ indicate the breathing mode of the tri-s-triazine ring units. The presence of broad bands from 3040–3300 cm^−1^ shows N–H and O–H stretching modes, which can be attributed to absorbed H_2_O molecules [6,21,47,53].

The broad peaks appearing at 3300–3000 cm^−1^ are due to the stretching vibrations of the O–H group on the TiO_2_ surface [19,54]. The peak located around 1626 cm^−1^ is due to bending vibrations resulting from chemically adsorbed H_2_O molecules [18]. The stretching vibrations of Ti–O and Ti–O–Ti exhibited by the TiO_2_ nanoparticles were observed at around 850 cm^−1^ [11,19,55]. The peaks at 1216, 1366, 1438, and 1738 cm^−1^ correspond to the carbonyl (C=O) mode. The peak observed at 1180 cm^−1^ corresponds to the stretching vibrations of the Ti–OH molecule [13]. In the g-C_3_N_4_/Ag–TiO_2_ sample, characteristic peaks attributed to both TiO_2_ and g-C_3_N_4_ were observed, confirming the presence of both materials in the composite sample [11,21].

### 3.3. Morphological Analysis

**Figure 5:**
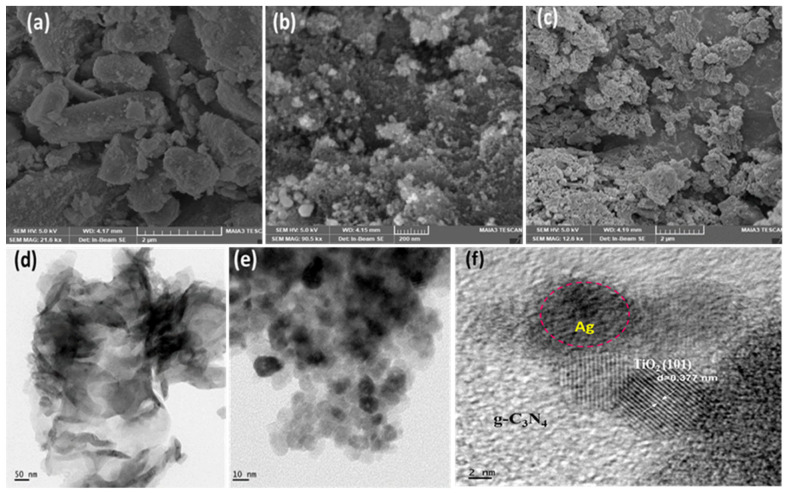
FE-SEM image of TiO_2_ (**a**), Ag–TiO_2_ (**b**), g-C_3_N_4_/Ag–TiO_2_ (**c**) and HR-TEM image of g-C_3_N_4_ (**d**), Ag–TiO_2_ (**e**), and g-C_3_N_4_/Ag–TiO_2_ (**f**).

Figure 5 shows the FE-SEM and HR-TEM morphologies of g-C_3_N_4,_ Ag–TiO_2_, and g-C_3_N_4_/Ag–TiO_2_ samples.

The SEM micrograph of pristine TiO_2_ nanoparticles exhibits irregularly shaped agglomerates, as shown in Figure 5a. The SEM and TEM images of Ag-doped TiO_2_ nanoparticles are shown in Figure 5b,e, respectively. In Figure 5b, the SEM image depicts the agglomeration-induced formation of a cluster of Ag–TiO_2_ nanoparticles and the homogeneous distribution of spherical Ag–TiO_2_ nanoparticles [56,57]. The presence of small Ag^0^ islands on the surface of Ag-doped TiO_2_ nanoparticles is clearly observed in the TEM image, as shown in Figure 5e [58]. The SEM image shows significant adhesion between the Ag–TiO_2_ nanoparticles and the g-C_3_N_4_ nanosheet, indicating a strong interface between the two constituents of the composite, as shown in Figure 5c. Notably, the presence of g-C_3_N_4_ improves the dispersion of TiO_2_ nanoparticles by preventing their agglomeration [11].

Based on the TEM image shown in Figure 5d, the g-C_3_N_4_ nanosheet exhibited a transparent appearance, which can be attributed to its thin and two-dimensional structure. These results also indicate that g-C_3_N_4_ has a fluffy structure [59].

The TEM image in Figure 5f depicts a strong interfacial region between g-C_3_N_4_ and Ag–TiO_2_. As can be seen in Figure 5f, the layered structure with the lightest color was identified as g-C_3_N_4_, whereas the stacked structure comprising small particles was inferred to be TiO_2_. The black particles situated between TiO_2_ and g-C_3_N_4_ were identified as Ag nanoparticles [38]. The lattice fringe spacing of TiO_2_ was 0.377 nm, which corresponds to the (101) crystal plane of TiO_2_ [42]. Therefore, it can be concluded that the TEM images of the g-C_3_N_4_/Ag–TiO_2_ nanocomposite represent the microstructure derived from its constituents, such as TiO_2_, g-C_3_N_4_, and Ag nanoparticles, thereby demonstrating the successful formation of the composite. Moreover, the results indicated that the agglomerated Ag–TiO_2_ nanoparticles were well dispersed on the porous architecture of g-C_3_N_4_. In addition, creating a close interfacial contact between g-C_3_N_4_ and Ag–TiO_2_ heterostructures may effectively promote the transfer of photoinduced electrons. This could potentially suppress the rate of charge–carrier recombination and enhance the overall photocatalytic activity [41,42].

### 3.4. Nitrogen Adsorption–Desorption Analysis

**Figure 6:**
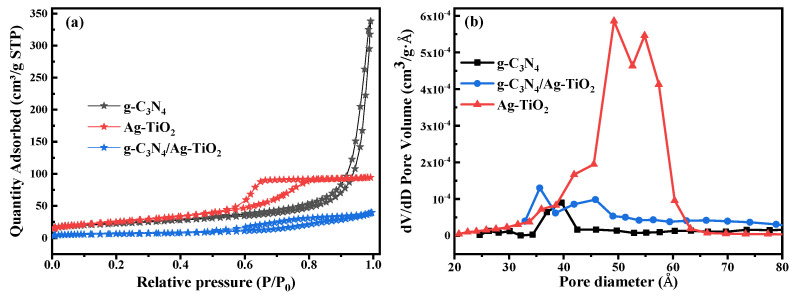
N_2_ adsorption–desorption isotherms (**a**,**b**) pore size distribution curves of the as-prepared samples.

Figure 6 depicts the nitrogen adsorption–desorption isotherms and pore size distribution of the as-synthesized samples.

Based on the N_2_ adsorption–desorption isotherms, all synthesized photocatalysts exhibited typical IV isotherms of mesoporous materials with H1, H2, and H3 hysteresis loops for g-C_3_N_4_, Ag–TiO_2_, and g-C_3_N_4_/Ag–TiO_2_, respectively, as shown in Figure 6a [17,21,43]. The specific surface area of Ag–TiO_2_ nanoparticles increased slightly because of the incorporation of Ag nanoparticles. However, the pore volume of Ag–TiO_2_ is not as high as its BET surface area because the Ag nanoparticles tend to block the pores in TiO_2_. Moreover, the surface area of the composite slightly decreased with the coupling of Ag–TiO_2_ to g-C_3_N_4_, potentially owing to the covering and blocking of the surface-active sites of Ag–TiO_2_ by g-C_3_N_4_ [21]. However, the excellent photocatalytic degradation efficiency of the g-C_3_N_4_/Ag–TiO_2_ nanocomposite might not be underestimated owing to the small specific surface area. In addition, the excellent photocatalytic performance of the composite is a consequence of a combination of factors such as the extended visible light absorption range, enhanced crystallite size, and reduced recombination of charge carriers owing to its narrowed bandgap energy [15]. Previous studies have reported that g-C_3_N_4_/Ag–TiO_2_ nanocomposites exhibit excellent photocatalytic performance despite having a smaller surface area than their constituent components [60].

Figure 6b shows the pore size distribution of the as-synthesized samples obtained using the Barrett–Joyner–Halenda method. The average pore sizes of the Ag–TiO_2_, g-C_3_N_4_, and g-C_3_N_4_/Ag–TiO_2_ samples were 6.5, 25.3, and 10.5 nm, with the corresponding total pore volumes of 0.15, 0.52, and 0.06 cm^3^·g^−1^, respectively. The g-C_3_N_4_ nanosheet showed the highest values for both pore size and total pore volume, which are essential factors in enhancing photocatalytic activity. The average crystallite size, energy bandgap, specific BET surface area (S_BET_), average pore size, and pore volume of the as-synthesized samples, including Ag–TiO_2_, g-C_3_N_4_, and g-C_3_N_4_/Ag–TiO_2_ nanocomposites, are summarized in Table 1.

### 3.5. Evaluation of Photocatalytic Activity

**Figure 7:**
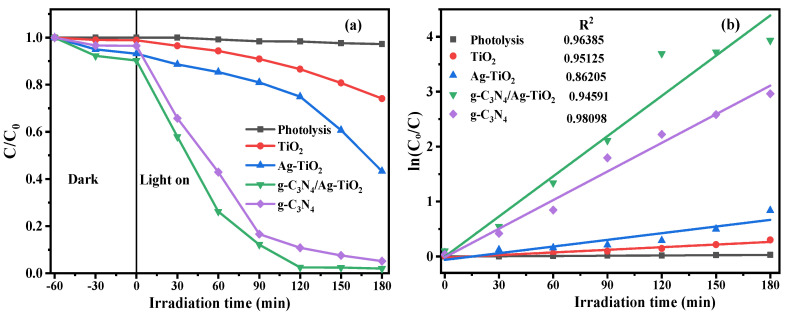
Photodegradation efficiency of RhB in the presence of photocatalysts under visible LED light irradiation (**a**,**b**) the corresponding pseudo-first-order kinetics of RhB degradation.

Figure 7 shows the photocatalytic degradation efficiency of RhB in the presence of photocatalysts under visible LED light irradiation.

The photolysis experiments were conducted in the absence of as-prepared photocatalysts, and almost no degradation of RhB dye was observed. The adsorption experiments were performed using the as-synthesized samples in the dark for 60 min. The g-C_3_N_4_/Ag–TiO_2_ nanocomposite exhibited an adsorption rate of 10%. In contrast, TiO_2_ exhibited the lowest adsorption rate of only 1.15%, whereas Ag–TiO_2_ and g-C_3_N_4_ had adsorption rates of 7% and 3.5%, respectively. The photocatalytic activities of all the prepared photocatalysts were evaluated by studying the photocatalytic decomposition efficiency of RhB dye under visible LED light irradiation. After 180 min of irradiation with visible LED light, g-C_3_N_4_ and g-C_3_N_4_/Ag–TiO_2_ photocatalysts exhibited remarkably higher photodegradation efficiencies of 94.83% and 98.04%, respectively, whereas pure TiO_2_ showed the lowest photodegradation efficiency of 25.9%. The binary Ag–TiO_2_ nanoparticles exhibited an intermediate photodegradation efficiency of 56.73%. The rate of degradation of binary Ag–TiO_2_ photocatalysts is higher than that of pristine TiO_2_ but lower than that of g-C_3_N_4_ and g-C_3_N_4_/Ag–TiO_2_ nanocomposites [48].

The enhanced photocatalytic activity of the g-C_3_N_4_/Ag–TiO_2_ composite was due to the synergetic effect of the localized surface plasmon resonance (LSPR) impact of Ag and its role as an electron-conduction bridge, as well as the existence of an excellent heterostructure between Ag–TiO_2_ and g-C_3_N_4_. These factors ameliorate the charge carrier separation efficiency and inhibit electron–hole pair recombination, which boosts the overall photocatalytic performance [38]. In terms of photogenerated charge separation, g-C_3_N_4_ has a more negative conduction band potential than that of TiO_2_. Meanwhile, the Ag nanoparticles serve as charge separation centers for photogenerated electrons from the conduction band of TiO_2_ owing to their lower Fermi level energy. Consequently, the photogenerated electrons and holes can be easily transported to the adjacent semiconductor surface, thus suppressing the rate of recombination of photoinduced electron–hole pairs, leading to improvements in photocatalytic efficiency [38,42].

Figure 7b shows the linear pseudo-first-order kinetics of the RhB dye degradation. The apparent reaction rate constants (k_app_) of TiO_2_, Ag–TiO_2_, g-C_3_N_4_, and g-C_3_N_4_/Ag–TiO_2_ were directly calculated by the equation ln(C/C_0_) = k_app_t, as shown in Figure 7b. The apparent rate constant (k_app_) of TiO_2_ nanoparticles is 1.66 × 10^−3^ min^−1^ because the catalytic activity of TiO_2_ is lower under visible light. The k_app_ of Ag–TiO_2_ is 4.65 × 10^−3^ min^−1^, which is mainly due to the plasmon resonance effect of the Ag nanoparticles on the surface of the TiO_2_ nanoparticles. The k_app_ of g-C_3_N_4_ is 16.46 × 10^−3^ min^−1^ and that of the g-C_3_N_4_/Ag–TiO_2_ nanocomposite is increased to 21.84 × 10^−3^ min^−1^. Therefore, the g-C_3_N_4_/Ag–TiO_2_ nanocomposite exhibited a reaction rate constant 13.16, 4.7, and 1.33 times higher than that of TiO_2_, Ag–TiO_2,_ and g-C_3_N_4_, respectively. The synergistic effect of the heterostructure comprising g-C_3_N_4_ nanosheets, TiO_2_, and Ag nanoparticles leads to enhanced photodegradation efficiency by effectively accelerating the transfer of photogenerated electrons and inhibiting the fast recombination of electron–hole pairs [17]. The photocatalytic degradation efficiency of g-C_3_N_4_/Ag–TiO_2_ nanocomposites was compared with that of various previous studies in Table 2. We found that the g-C_3_N_4_/Ag–TiO_2_ nanocomposite fabricated in this study demonstrated excellent photocatalytic activity compared to those in previous works.

### 3.6. Mechanism of Photocatalytic Degradation

**Figure 8:**
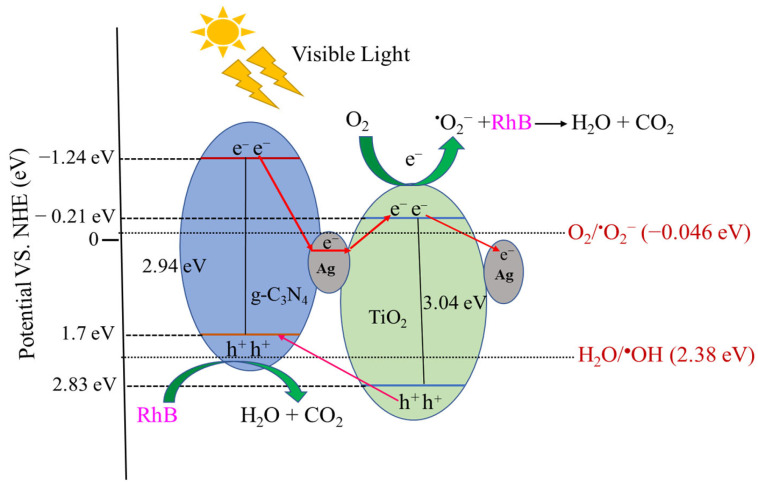
Schematic illustration of proposed photodegradation mechanism of heterostructured g-C_3_N_4_/Ag–TiO_2_ nanocomposite photocatalyst under visible LED light illumination.

Based on the results and discussions presented, including XRD, UV/vis DRS, and PL analyses, as well as previous reports [11,15,17,38,41], a plausible photodegradation mechanism for ternary g-C_3_N_4_/Ag–TiO_2_ photocatalysts is proposed in Figure 8. The conduction band (E_CB_) and valence band (E_VB_) potentials relative to the normal hydrogen electrode (NHE) of the samples were determined using the following equations:

(5) $$E_{VB}=\chi -E^{e}+{0.5E}_{g}$$

(6) $$E_{CB}=E_{VB}-E_{g}$$

where χ is the absolute electronegativity of the semiconductor; TiO_2_ = 5.81 eV and g-C_3_N_4_ = 4.73 eV. The energy of free electrons on the hydrogen scale is denoted as E^e^ and has a value of 4.5 eV, and E_g_ is the bandgap energy [42,43]. From UV/vis DRS analysis results, the valence band and conduction band potentials of g-C_3_N_4_ were 1.7 eV and −1.24 eV, while those of TiO_2_ were 2.83 eV and −0.21 eV, respectively.

Figure 8 shows that under visible LED light irradiation, electrons were excited from the valence band to the conduction band (CB) of TiO_2_ and g-C_3_N_4_. Electrons could easily migrate from g-C_3_N_4_ to TiO_2_ because of the more negative conduction band potential of g-C_3_N_4_ (E_VB_ = −1.24 eV) than that of TiO_2_ (E_VB_ = −0.21 eV). These electrons combine with the adsorbed O_2_ on the TiO_2_ surface to form ^•^O_2_^−^, which is then used for the decolorization of RhB. Similarly, it appears that g-C_3_N_4_ (+1.7 V vs. NHE) has a lower valence band potential than H_2_O/^•^OH (+2.38 V vs. NHE), indicating that holes are incapable of oxidizing H_2_O into ^•^OH radical species; however, they can directly participate in RhB oxidation. Therefore, holes and ^•^O_2_^−^ radicals may play a major role in the decolorization of RhB into harmless byproducts such as H_2_O and CO_2_ [17,42,43,61].

Ag deposited on TiO_2_ in g-C_3_N_4_/Ag–TiO_2_ nanocomposites acts as a bridge for electron conduction, resulting in improved separation of electron–hole pairs on g-C_3_N_4_ through the generation of a Schottky barrier between Ag nanoparticles and TiO_2_ [17,38]. In addition, due to the surface plasma resonance effect, Ag nanoparticles in heterostructured g-C_3_N_4_/Ag–TiO_2_ nanocomposites can be photoexcited, producing electrons that migrate to the conduction band of TiO_2_ [48]. The transfer of excess electrons from the conduction band of TiO_2_ to Ag nanoparticles in heterostructured g-C_3_N_4_/Ag–TiO_2_ nanocomposites reduces the recombination of photoinduced charge carriers [15]. The incorporation of Ag nanoparticles, in addition to the excellent heterostructure formation between TiO_2_ and g-C_3_N_4,_ results in excellent photocatalytic degradation efficiency of RhB [17,38]. Moreover, the close interfacial connections between Ag–TiO_2_ and g-C_3_N_4_ allow for efficient electron migration and spatial separation of electrons and holes, retarding their recombination rate and thereby improving photoactivity [60].

## 4. Conclusions

A simple hydrothermal technique followed by calcination was used to fabricate g-C_3_N_4_/Ag–TiO_2_ composites with high photocatalytic performance for RhB dye degradation under visible light irradiation. The heterostructured g-C_3_N_4_/Ag–TiO_2_ composite was synthesized under optimal conditions at 180 °C for 6 h with a 3:1 weight ratio of g-C_3_N_4_ to Ag–TiO_2_. Various analytical tools were employed to characterize the crystal structures, morphologies, microstructures, chemical structures, and optical and physicochemical properties of the as-synthesized samples. The experimental results demonstrate that the heterostructured g-C_3_N_4_/Ag–TiO_2_ composite is outstanding for the decomposition of RhB dye under visible LED light irradiation and exhibits a degradation efficiency of 98.04%. The high photocatalytic activity observed for the g-C_3_N_4_/Ag–TiO_2_ nanocomposite may be due to the synergetic effect of the LSPR effect of Ag and its role as an electron-conduction bridge, as well as the formation of a strong heterostructure between Ag–TiO_2_ and g-C_3_N_4_. These factors significantly enhanced the efficiency of photogenerated charge carrier separation and transfer, improved visible light absorption, increased crystallite size, and inhibited charge carrier recombination, thereby improving the overall photocatalytic performance. Therefore, the present study shows that g-C_3_N_4_/Ag–TiO_2_ nanocomposites possess enormous potential for the effective elimination of hazardous organic pollutants in wastewater.

## Figures and Tables

**Table 1:** Crystallite size, energy bandgap (E_g_), specific surface area (S_BET_), pore size, and pore volume of the as-synthesized samples.

Photocatalysts	Crystallite Size (nm)	E_g_ (eV)	S_BET_ (m^2^·g^−1^)	Pore Size (nm)	Pore Volume (cm^3^·g^−1^)
Ag–TiO_2_	9.1	2.89	90.8	6.5	0.15
g-C_3_N_4_	4.3	2.94	79.5	25.3	0.52
g-C_3_N_4_/Ag–TiO_2_	7.6	2.73	22.5	10.5	0.06

**Table 2:** Comparison of photocatalytic performance of g-C_3_N_4_/Ag–TiO_2_ nanocomposites for degrading RhB dye obtained from the current study versus previous studies.

**Photocatalyst**	Catalyst Dosage	**Pollutant Concentration**	**Light Source** **(λ ≥ 420 nm)**	**Irradiation Time**	Degradation Efficiency	**Refs.**
g-C_3_N_4_/Ag–TiO_2_	50 mg	RhB, 10 mg/L	300 W Xe lamp	30 min	98.13%	[42]
g-C_3_N_4_/Ag–TMCs	20 mg	RhB, 20 mg/L	300 W Xe lamp	15 min	100%	[15]
g-C_3_N_4_/Ag–TiO_2_	50 mg	RhB, 5 mg/L	500 W Xe lamp	105 min	92.7%	[17]
g-C_3_N_4_/Ag–TiO_2_	50 mg	RhB,10 mg/L	300 W Xe lamp	60 min	100%	[43]
g-C_3_N_4_/Ag–TiO_2_	50 mg	RhB, 5 mg/L	300 W Xe lamp	30 min	96%	[41]
Ag/g-C_3_N_4_/TiO_2_	20 mg	RhB, 10 mg/L	300 W Xe lamp	120 min	100%	[38]
g-C_3_N_4_/Ag–TiO_2_	50 mg	RhB, 10 mg/L	50 W LED lamp	180 min	98.04%	This work

## Data Availability

The data that support the findings of this study are available upon reasonable request from the corresponding author.
